# Woman With a Swollen Finger

**DOI:** 10.1016/j.acepjo.2025.100081

**Published:** 2025-02-20

**Authors:** Gayle Galletta

**Affiliations:** Department of Emergency Medicine, UMass Chan Medical School, Worcester, Massachusetts, USA

**Keywords:** hydroxyapatite crystal deposition disease, arthropathy, HADD

## 1 Case Presentation

**Figure 1:**
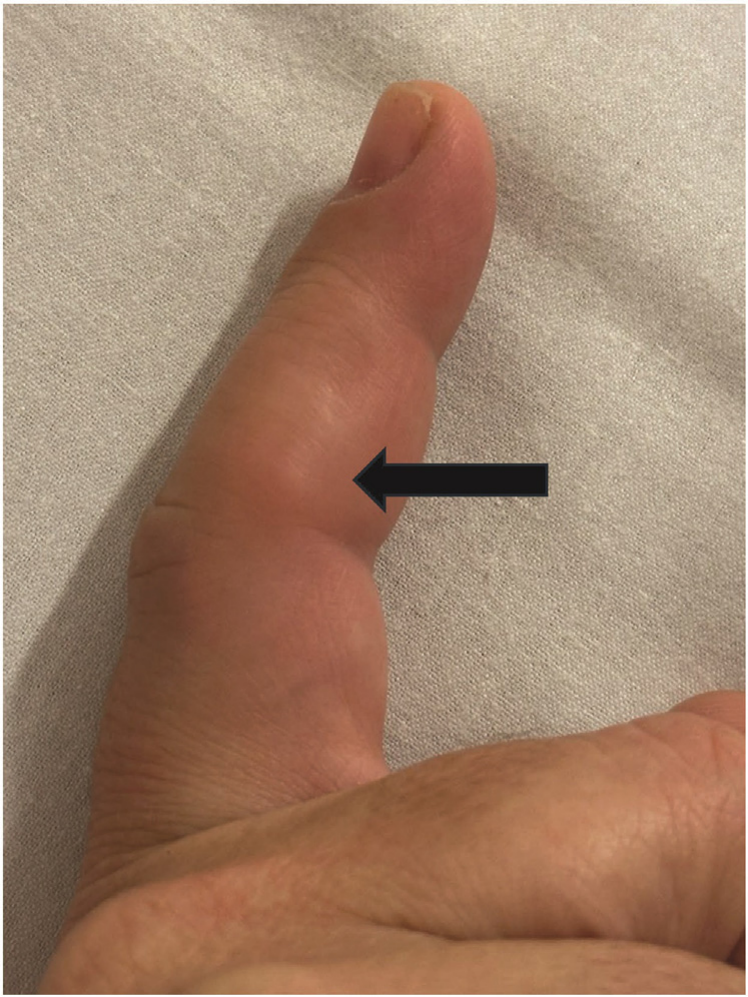
Right index finger with tender nodule (black arrow) at the proximal interphalangeal joint.

**Figure 2:**
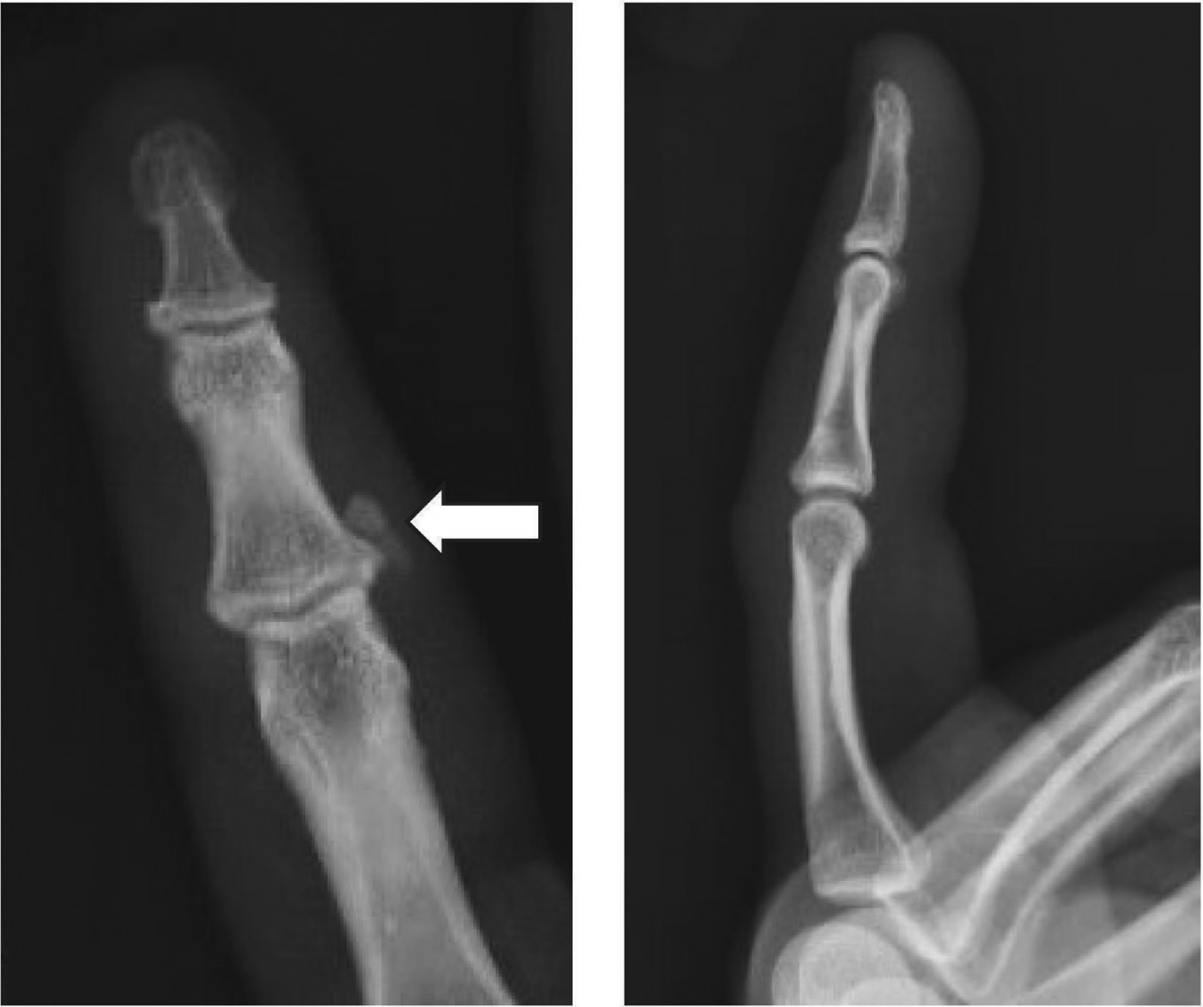
Anterior-posterior and lateral images of the right index finger demonstrate a soft tissue calcification at the ulnar aspect of the index finger proximal interphalangeal joint (white arrow), suggestive of calcium hydroxyapatite deposition disease.

A 46-year-old right-handed woman presented to a community emergency department with a 2-day history of progressively worsening pain, redness, and swelling to her right index finger proximal interphalangeal joint (Fig 1). She denied trauma but states that the pain worsens with activity, especially working as a barista at a coffee shop. The physical examination was normal except for redness, swelling, and exquisite tenderness of the ulnar aspect of the right index finger proximal interphalangeal joint. There was no tenderness over the flexor tendon. Finger x-rays were obtained (Fig 2).

## 2 Diagnosis: Hydroxyapatite Crystal Deposition Disease

Hydroxyapatite crystal deposition disease (HADD) is a rare cause of hand and wrist pain and causes swelling that may mimic infectious arthritis or tenosynovitis.^1^ The diagnosis may be made by the classic history of sudden onset of atraumatic pain and classic x-ray findings.^2^ The x-ray calcifications seen with HADD are not present with tenosynovitis.^3^ Treatment includes nonsteroidal anti-inflammatories and a splint for comfort.^1^^,^^3^ Symptoms typically improve within 4 to 7 days and resolve within 4 weeks.^1^ HADD has a high rate (up to 70%) of misdiagnosis, and many patients undergo unnecessary workups and surgical treatment.^3^

## Funding and Support

By *JACEP Open* policy, all authors are required to disclose any and all commercial, financial, and other relationships in any way related to the subject of this article as per ICMJE conflict of interest guidelines (see www.icmje.org). The authors have stated that no such relationships exist.

## Conflict of Interest

The author works as a paid medical consultant for Defibrio, a defibrillator start-up company. The author’s consulting work had no influence on this manuscript.

## References

[1] Bernier D., Marteau E., Roulet S. (2021). Hydroxyapatite deposits of the hand and wrist: a diagnosis not to be ignored. Pan Afr Med J.

[2] Dimmick S., Hayter C., Linklater J. (2022). Acute calcific periarthritis-a commonly misdiagnosed pathology. Skeletal Radiol.

[3] Doumas C., Vazirani R.M., Clifford P.D., Owens P. (2007). Acute calcific periarthritis of the hand and wrist: a series and review of the literature. Emerg Radiol.

